# Regional heterogeneity impacts gene expression in the subarctic zooplankter *Neocalanus flemingeri* in the northern Gulf of Alaska

**DOI:** 10.1038/s42003-019-0565-5

**Published:** 2019-09-02

**Authors:** Vittoria Roncalli, Matthew C. Cieslak, Martina Germano, Russell R. Hopcroft, Petra H. Lenz

**Affiliations:** 1 Pacific Biosciences Research Center, University of Hawai’i at Mānoa, 1993 East-West Rd., Honolulu, HI 96822 USA; 2 Department of Genetics, Microbiology and Statistics, Facultat de Biologia, IRBio, Universitat de Barcelona, Av. Diagonal 643, 08028 Barcelona, Spain; 3 Institute of Marine Science, University of Alaska, Fairbanks, 120 O’Neill, Fairbanks, AK 99775-7220 USA

**Keywords:** Gene expression, Ecophysiology, Molecular ecology, Functional genomics

## Abstract

Marine pelagic species are being increasingly challenged by environmental change. Their ability to persist will depend on their capacity for physiological acclimatization. Little is known about limits of physiological plasticity in key species at the base of the food web. Here we investigate the capacity for acclimatization in the copepod *Neocalanus flemingeri*, which inhabits the Gulf of Alaska, a heterogeneous and highly seasonal environment. RNA-Seq analysis of field-collected pre-adults identified large regional differences in expression of genes involved in metabolic and developmental processes and response to stressors. We found that lipid synthesis genes were up-regulated in individuals from Prince William Sound and down-regulated in the Gulf of Alaska. Up-regulation of lipid catabolic genes in offshore individuals suggests they are experiencing nutritional deficits. The expression differences demonstrate physiological plasticity in response to a steep gradient in food availability. Our transcriptional analysis reveals mechanisms of acclimatization that likely contribute to the observed resilience of this population.

## Abstract (web summary)

Vittoria Roncalli et al. used transcriptomics to study the acclimatization of a pre-adult copepod to the highly variable environment of the northern Gulf of Alaska. They find differences in expression of metabolic genes, particularly those involved in lipid metabolism, in different regions of the Gulf.

## Introduction

Over the past 50 years, large-scale latitudinal shifts in communities have been documented in both terrestrial and aquatic ecosystems^1–4^. While community regime shifts are correlated with an overall increase in average temperatures, the proximate causes for observed changes are more complex: climate forcing leads to cascading effects that alter many abiotic and biotic factors, which in turn affect individual fitness and create new sets of winners and losers^5,6^. However, some communities appear to be resilient to climate variability and predicting how communities might respond to change is an active area of investigation^1,7,8^.

One approach has been to investigate adaptive capacity of key species to stressors associated with global climate change, such as increases in temperature and ocean acidification^9,10^. Species with genetic divergence among populations have the potential for rapid evolution and resilience to climate change through natural selection acting on existing genetic variation, and immigration of resilient genotypes following local extinction events^11,12^. However, replacement with novel genotypes may be less available to planktonic species, which are not only widely distributed, but also in constant motion as they drift over large distances within oceanic currents^13,14^. Oceanic mesozooplankton, like copepods show little genetic differentiation within oceanic provinces, and sometimes even across multiple oceans^15–17^. Thus, these organisms may depend on phenotypic plasticity to adapt to a heterogeneous and changing environment.

Planktontic organisms can experience nonoptimal conditions through much of their life in oceanic habitats where they can be advected over large distances^15,18,19^. How do these organisms compensate for suboptimal conditions physiologically, while maintaining their fitness? High-throughput sequencing technologies are providing new opportunities to investigate this question in zooplankton. Transcriptional differences among individuals can be quantified using RNA-Seq. Environment-mediated shifts in gene expression have been well documented both in the laboratory and in the field^20–22^. Relative gene expression in combination with a functional analysis of the regulated genes can inform how an organism is responding to its ambient environment. Here, we used this approach to examine the transcriptional physiology of a marine zooplankter, the copepod *Neocalanus flemingeri*, a high-latitude species with a complex life history adapted to optimize synchronization with a seasonally changing environment that is also spatially heterogeneous.

The northern Gulf of Alaska is characterized by steep gradients in the physical and chemical environments driven by patterns in circulation, salinity, winds, and macro- and micro-nutrients^23^. Differences in the physical and chemical environment lead to resource gradients across the shelf, which impact the community composition and abundances of phytoplankton, microzooplankton, and mesozooplankton^18,19,24^. In this highly variable environment, *N. flemingeri* is a biomass dominant in April–May throughout the region from Prince William Sound to the outer shelf of the Gulf of Alaska^25^. Here, we applied RNA-Seq technology to obtain global gene expression profiles of pre-adult *N. flemingeri* collected across the shelf and in Prince William Sound. Large variation in transcriptional physiology was observed among pre-adult *N. flemingeri* collected across the shelf and in Prince William Sound. Regional differences in the expression of genes associated with metabolism, response to stress and development were consistent with gradients in chlorophyll *a*, which is an indicator of food levels. While the results indicate a large capacity for physiological acclimatization in *N. flemingeri*, they also suggest that offshore individuals in the high-nutrient low-chlorophyll (HNLC) region of the Gulf of Alaska were experiencing nutritional stress, thus interfering with lipid accumulation required for successful preparation for diapause.

## Results

### Study overview

**Fig. 1:**
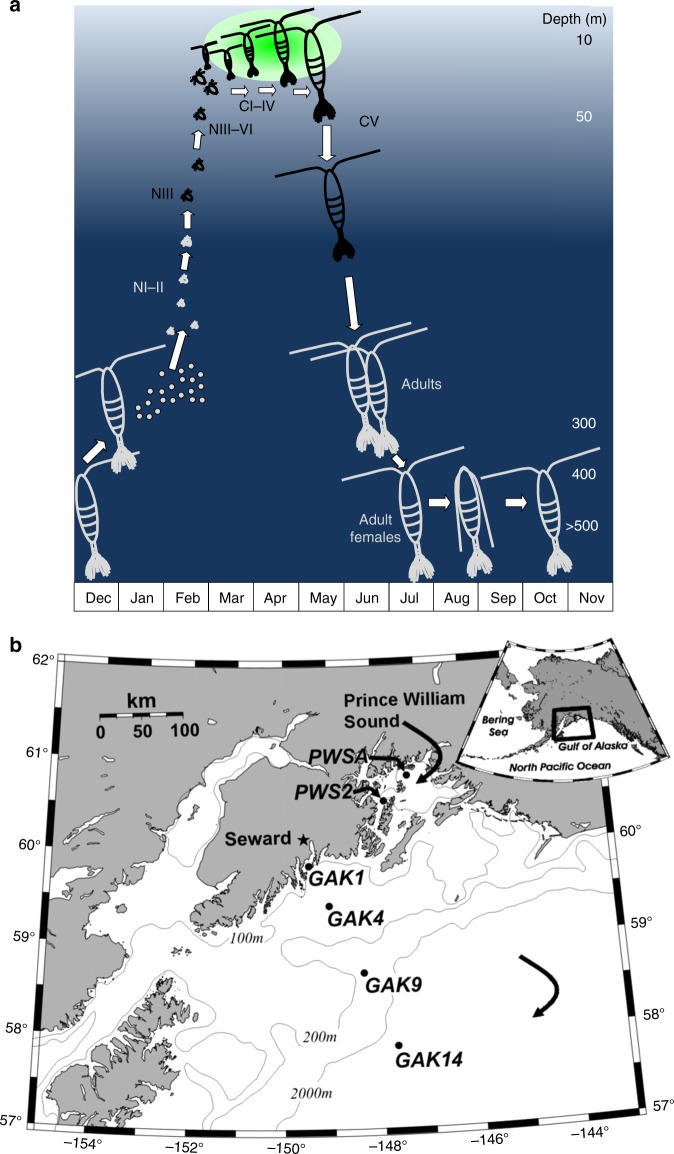
*Neocalanus flemingeri* life cycle and map of study site. **a** Diagram of the life cycle of *N. flemingeri* from December to November. Feeding stages (nauplii [NIII-NVI] and copepodids [CI-CV]), are shown in black, nonfeeding stages (adult females and males [CVI] and early nauplii [NI and NII]) in light gray. Green oval indicates period of increased production starting in late March/early April and approximate timing of phytoplankton bloom (late April—early/mid May). Depths in meters (not to scale) indicated on the right. Modified from Lenz and Roncalli^26^. **b** Map showing locations and names of stations in Prince William Sound (PWS) and Gulf of Alaska (GAK). Curved arrows: Prince William Sound (top); off shelf Gulf of Alaska (bottom; depth > 2000 m). Light gray lines indicate depth contours. Inset shows map of Alaska with the location of sampling area indicated by the black box

*Neocalanus flemingeri* were collected in early May, when most individuals are in the pre-adult developmental stage (copepodid CV) and preparing for diapause, which requires the accumulation of storage lipids (Fig. 1a)^26^. Collections occurred during a 1-week oceanographic cruise (early May 2015) from six locations spanning the inner to outer shelf along the Seward Line in the northern Gulf of Alaska and two stations within Prince William Sound (Fig. 1b and Table 1). Gene expression profiles were obtained for individual pre-adults (CV) collected at each station (*n* = 18) using RNA-Seq (Supplementary Table 1). Functional analysis of gene expression patterns was compared with genetic distance between individuals from different stations and with environmental gradients.

**Table 1:** Summary of spring 2015 collections of *Neocalanus flemingeri* copepodid stage CV individuals analyzed for whole-organism patterns of gene expression using RNA-Seq

	PWS2	PWSA	GAK1	GAK4	GAK9	GAK14
Date	May 7, 2015	May 7, 2015	May 10, 2015	May 10, 2015	May 6, 2015	May 5, 2015
CTD cast#	22	24	34	40	10	7
Time (h)	15:10	19:30	07:45	14:15	08:30	18:00
Latitude N	60° 32.1′	60° 49.3′	59° 50.7′	59° 24.5′	58° 40.8′	57° 56.6
Longitude W	147° 48.2′	147° 24′	149° 28′	149° 2.9′	148° 21′	147° 39′
Depth (m)	738	476	264	199	276	2720

CTD (conductivity, temperature, and depth) cast#: date and time of CTD cast (Alaska daylight time), latitude and longitude, and total depth are listed for each station. Collection of zooplankton by vertical net tow from 100 to 0 m at all stations

### Environmental gradients in the northern Gulf of Alaska

**Fig. 2:**
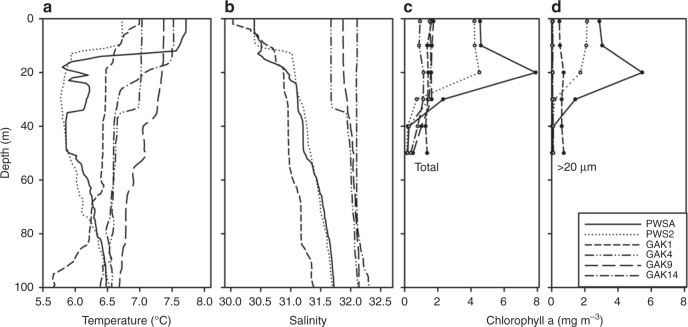
Environmental profiles measured at sample locations within Prince William Sound and the Gulf of Alaska. **a** Temperature (°C); **b** Salinity; **c** Total chlorophyll *a* (mg m^−3^); **d** Chlorophyll *a* (mg m^−3^) retained on 20 µm filters. Stations identified by line patterns indicated in D (inset)

**Fig. 3:**
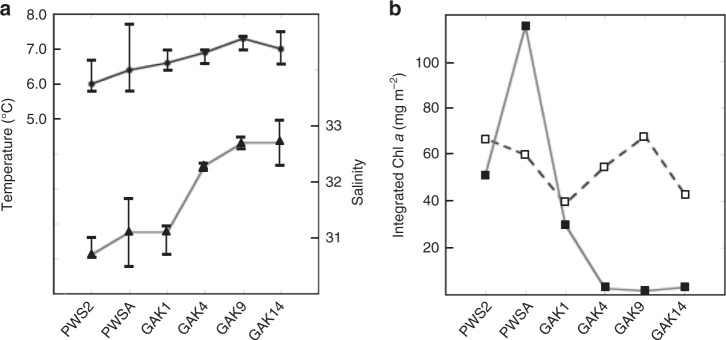
Comparison of average temperature, salinity and chlorophyll *a* across sampling stations. **a** Temperature and salinity measured by CTD and averaged over the upper 50 m. Error bars indicate range measured at station between 0 and 50 m. **b** Integrated chlorophyll *a* (mg m^−2^) from surface to 50 m in two size fractions: <20 µm (open squares) and >20 µm (filled squares)

Concurrent environmental monitoring revealed differences in the vertical structure of temperature and salinity across stations consistent with previously described inshore–offshore gradients (Fig. 2a, b). All stations showed some stratification caused by temperature and/or salinity gradients, with the most shallow and pronounced gradients (~10 m) occurring at PWSA and PWS2. Average temperatures between 1 and 50 m ranged between 6 and 7.3 °C (Fig. 3a). Temperatures at 100 m were similar across stations (6.5 °C) with the exception of GAK1 (<6 °C). The low temperature at GAK1 correlated with the lowest salinities measured at this depth (Fig. 2b). In general, surface salinities were lower at nearshore stations (PWS2, PWSA, and GAK1) as would be expected from the greater influence of freshwater inputs at these stations. Salinity increased with distance from shore as shown by the GAK4, GAK9, and GAK14 conductivity profiles and average salinities in the upper 50 m across the six stations (Figs. 2b and Fig. 3a).

Chlorophyll *a* levels varied across stations (Fig. 2c, d). Highest chlorophyll *a* concentrations were measured in the upper 30 m in Prince William Sound. Chlorophyll *a* levels declined with distance from shore along the Seward Line. While the majority of chlorophyll was in large cells within PWS, it was split between large and small cells at GAK1, and it was mostly in small cell sizes at the remaining GAK stations. Large-cell chlorophyll was very low at GAK4, GAK9, and GAK14 (Fig. 2d). This pattern is even more apparent in the integrated chlorophyll *a* values shown separately for large and small cells across stations (0–50 m, Fig. 3b)—chlorophyll *a* concentrations of small cells were similar across stations, while large-cell chlorophyll *a* differed by more than an order of magnitude (2 vs. >100 mg chl*a* m^−2^) between PWS and the offshore stations.

### Individuals segregated by station based on gene expression

**Fig. 4:**
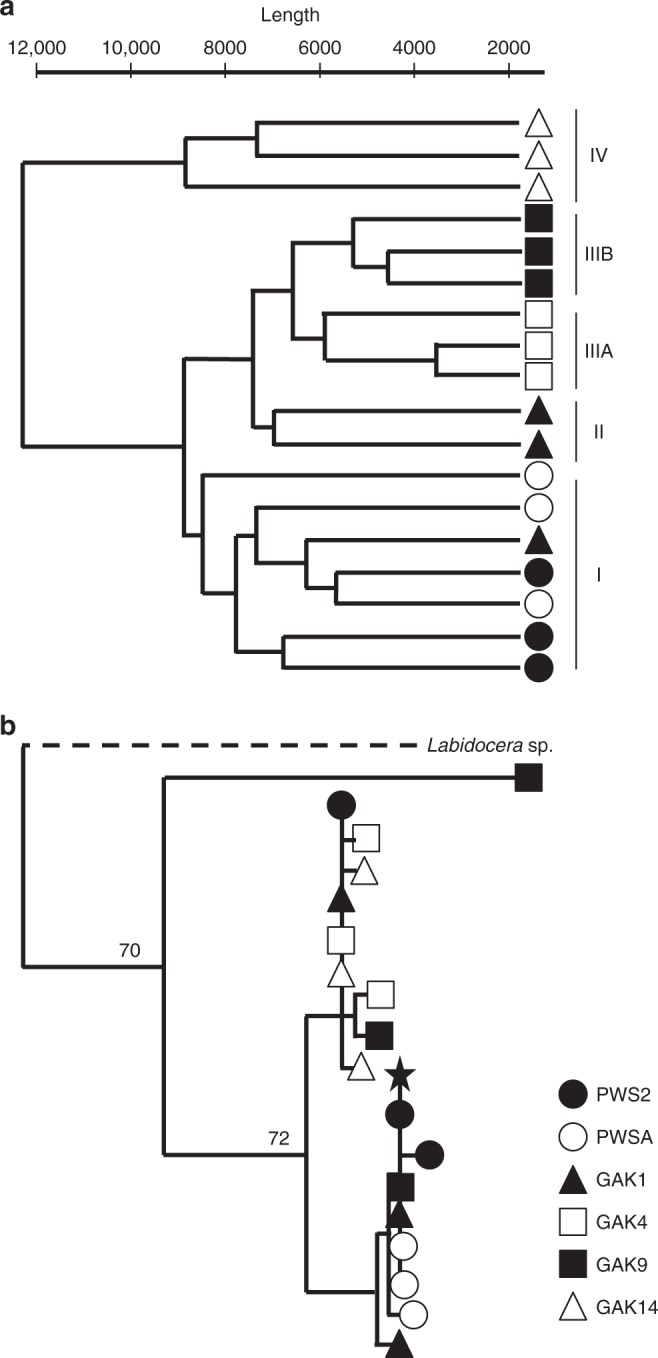
Cluster analyses by gene expression of all transcripts and genetic distance for mtCOI genes in *Neocalanus flemingeri* CV individuals collected at six stations (Fig. 1b). **a** Dendrogram of all CV individuals (*n* = 18) obtained by hierarchical cluster analysis based on relative expression of 51743 genes showed four major groups (I–IV). Each line represents a single CV individual. Symbols indicate collection station (see legend in figure). Length of the *y*-axis indicates distance between clusters and individuals. The GAK1-S83 individual, used to generate the reference transcriptome clustered with another GAK1 individual in group II. **b** Cladogram of the mtCOI haplotypes of the same individuals (see symbols in legend above). For reference, *a Labidocera* sp. haplotype (Acc. No. KC594137)^59^ (outgroup, dashed line) and the *N. flemingeri* small form haplotype (*) (Acc. No. AB526881)^55^ are also shown. Bootstrap support values are indicated. The tree is unrooted

Agnostic clustering of individuals by gene expression profiles indicated regional and station differences. The *N. flemingeri* CVs segregated into four major groups with greater similarity found among individuals from the same station (Fig. 4a). Group I included all individuals from the two PWS stations (6) and one individual from GAK1 (Fig. 4a). Group II included the remaining individuals from GAK1 (2) and group III separated individuals from two stations (GAK4 and GAK9) into station-specific subgroups. Group IV included all  three individuals from station GAK14.

The station/region-specific clustering observed in Fig. 4a did not correlate with genetic divergence. Genetic variation among stations contributed to ≤18% of the variance, while variance within station contributed ≥80% for the three mitochondrial and the nuclear 18S markers (Supplementary Table 2). A cladogram of the mtCOI haplotypes underscores the lack of regional differences in haplotypes (Fig. 4b). With the exception of a single individual (GAK9), the same haplotypes were present at stations across the study site. While this GAK9 individual clustered with the other two individuals with respect to the gene expression profile (Fig. 4a), the percentage of short sequence reads mapped against the reference transcriptome was lower than that of all other individuals (82 vs. 89–95%; Supplementary Fig. 1). As a result, this individual was removed from all downstream analyses.

### Large-scale differences in gene expression among stations

Overall, 6472 genes were identified as differentially expressed among the six stations (GLM, *p* ≤ 0.05 after FDR correction). Pairwise  comparisons between stations (glmLRT, likelihood ratio test) confirmed the regional and station differences shown in the cluster analysis (Fig. 4a). The lowest number of differentially expressed genes (DEGs) was found in the comparisons between individuals from the two PWS stations (DEGs = 526) and between the two more offshore stations (GAK9 and GAK14; DEGs = 714) (Supplementary data 1). In contrast, the number of DEGs between PWS2 and GAK4, and PWS2 and GAK14 exceeded 2500 (Supplementary data 1).

Three major conserved eukaryotic processes: ‘cellular process’ [GO:0009987], ‘metabolic process’ [GO:0008152], and ‘biological regulation’ [GO:0065007] were represented among 3107 DEGs annotated with GO terms (E-value cutoff 1e−05). Other biological processes among the DEGs included ‘response to stress’ [GO:0006950] and ‘developmental process’ [GO:0032502], as well as several processes with more specific functions (higher level of organization) such as ‘localization’ [GO:0051179] and ‘immune system process’ [GO:006955] (Supplementary Fig. 2).

**Fig. 5:**
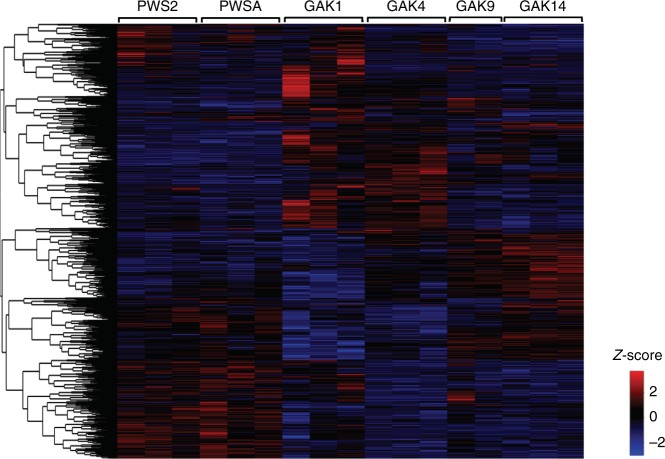
Heatmap showing patterns of gene expression in *Neocalanus flemingeri* CVs from six locations (*n* = 17). Relative expression shown as *z*-scores of differentially expressed genes (*n* = 3107) with annotations. Each column indicates an individual CV aggregated by station as shown by brackets on top. Relative expression is color coded by the magnitude of differential expression between expression level for each individual and mean expression across all individuals in log_2_(RPKM+1) normalized by the variance (scale bottom right). Genes were ordered by similarity of expression pattern as shown by the dendrogram (left). DEGs were identified by GLM test with *p* ≤ 0.05 after FDR correction. Data for figure with transcript identifications and annotations are available in Supplementary data 4

Relative expression of the annotated DEGs (*n* = 3107) is shown in a heatmap of *z*-scores (Fig. 5). The pattern highlights individual and regional differences in gene expression, especially between PWS and the two more offshore Seward Line stations (GAK9 and GAK14). Furthermore, a large number of genes appear to be upregulated in GAK1 and GAK4 individuals only. A smaller group of upregulated and downregulated genes in GAK1 individuals was more similar to PWS individuals than to those from GAK4. A heatmap of *z*-scores of all transcripts that did not annotate at an E-value of 1e−05 or lower (*n* = 3365) showed a similar pattern of station and regional differences in expression (Supplementary Fig. 3).

**Fig. 6:**
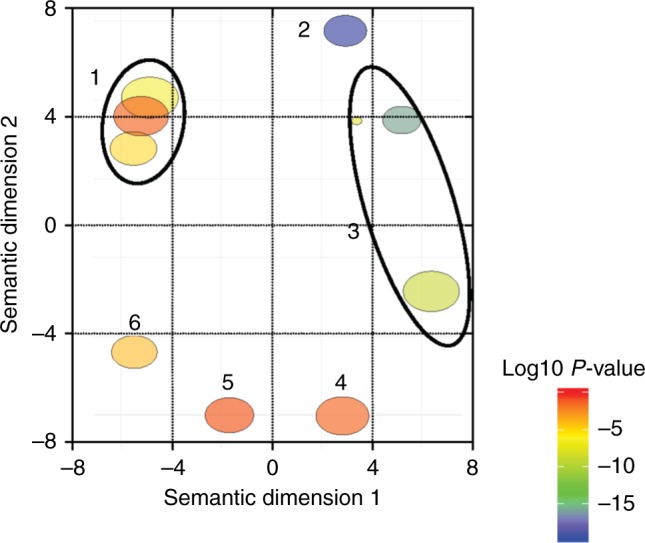
ReviGO semantic analysis of enriched GO terms among differentially expressed genes. DEGs annotated with GO terms and identified in pairwise comparisons between all stations (glmLRT, 15, Supplementary data 1) were independently tested for enriched processes against the 24356 annotated transcripts in the GAK1 reference transcriptome using TopGO (see Methods). All enriched GO terms were combined and summarized by functional similarity using reviGO. GO terms with a shared GO parent have been circled (black line). Bubble annotation: (1) ‘lipid metabolic process’ [GO:0006629, GO:0006869, GO:0006685]; (2) ‘glutathione metabolic process’ [GO:0006749]; (3) ‘response to stress’ [GO:0006950, GO:0006974, GO:0006955]; (4) ‘multicellular organism development’ [GO:0007275]; (5) ‘protein ubiquitination’ [GO:0016567]; (6) ‘purine nucleotide metabolic process’ [GO:0006163]

Enrichment analysis of the annotated DEGs, identified from pairwise comparisons (glmLRT) (15, DEGs = 3107) was followed by reviGO summarization analysis. Significant differences among stations (GLM, *p* value ≤ 0.05) included transcripts involved in ‘lipid metabolic process’ [GO:0006629], ‘response to stress’ [GO:0006950] and ‘multicellular organism development’ [GO:0007275] (Fig. 6 and Supplementary data 2). At a higher level of organization, ‘lipid transport’ [GO:0006869] and ‘sphingomyelin catabolic process’ [GO:0006685] were enriched within the broader category of lipid metabolic process. Within ‘response to stress’ [GO:0006950], ‘cellular response to DNA damage stimulus’ [GO:0006974] and ‘immune system process’ [GO:0006955] were enriched in 10 out of 15 likelihood pairwise comparisons (Supplementary data 2). Other biological processes that were overrepresented among the DEGs included ‘protein ubiquitination’ [GO:0016567], ‘glutathione metabolic process’ [GO:0006749] and ‘purine nucleotide metabolic process’ [GO:0006163], which were enriched in 4 out of 15 likelihood pairwise comparisons (Fig. 6 and Supplementary data 2).

### Drivers of station-specific gene expression differences

#### Metabolism

**Fig. 7:**
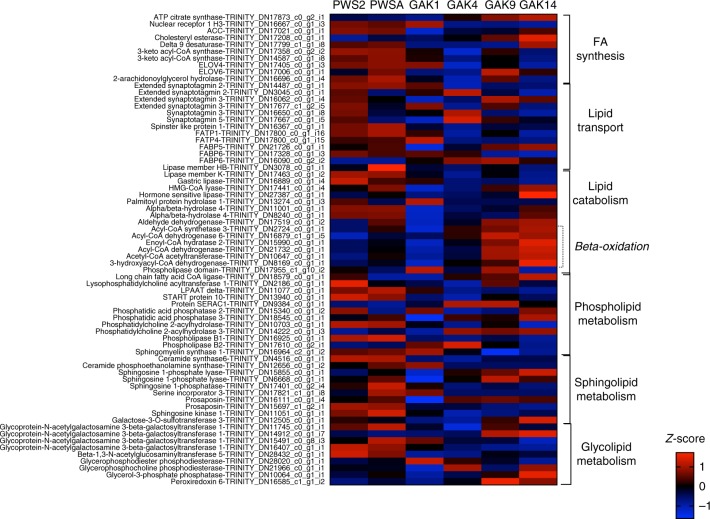
Pattern of expression of genes involved in lipid metabolism in *Neocalanus flemingeri* CVs. Heatmap of differentially expressed genes (DEGs) that annotated to GO terms involved in ‘lipid metabolic process’ [GO:0006629] and ‘lipid transport’ [GO:0006869]. Color coding for each gene (scale bottom right) indicates the magnitude of differential expression among stations calculated as the mean *z*-score averaged for individuals from that station (*n* = 3, except for GAK9 *n* = 2). Station identified above each column. Genes were grouped by function as indicated by the brackets on the right. Gene annotations and their Trinity ID number are listed on the left. Annotation *E*-values for this set of genes ranged from 1e−07 to 1e−180 (Supplementary data 4)

We investigated expression patterns of specific groups genes involved in processes, such as lipid and protein metabolism, since the enrichment analysis indicated large regional differences in expression. Lipid metabolism was an overrepresented process among the DEGs in 12 out of 15 pairwise comparisons (glmLRT) (Fig. 6 and Supplementary data 2). A total of 70 annotated DEGs were identified as involved in phospholipid, fatty acid (FA), glycerophospholipid and sphingolipid metabolism (Fig. 7). The most common functional categories included: (1) lipid synthesis, which is the process by which food resources are converted into FAs; (2) lipid catabolism, which is the use of lipids to support current energy needs; and (3) lipid transport. Within the context of diapause preparation, lipid synthesis is a key metabolic process: genes that regulate fat accumulation are upregulated in insects^27^. In the copepod, relative expression of genes involved in lipid metabolism differed among stations and ranged from the upregulation of transcripts encoding lipid synthesis enzymes to the upregulation of transcripts encoding enzymes involved in lipid catabolism.

Genes grouped by function highlight how relative expression varied by station and region (Fig. 7). Within the broader category of FA synthesis, one acyl-CoA carboxylase (*ACC*), one FA desaturase (*delta 9*), and two keto-acyl-CoA synthases (*KCS1*) were upregulated in PWS individuals (PWS2 and PWSA) compared with individuals from the GAK stations (GAK4, GAK9, and GAK14) (Fig. 7). Relative expression of these genes in GAK1 individuals was often intermediate (Fig. 7). Two genes annotated as lipid elongases (*ELOV4* and *ELOV6*) were upregulated in PWS individuals. *ELOV4* was also upregulated in GAK1 individuals. Phospholipid, sphingolipid, and glycolipid synthesis genes were more highly expressed in PWS than in GAK4, GAK9, and GAK14 individuals. Upregulation of several ceramide synthases, sphingosine phosphatases, and glycoprotein-N-acetylgalactosamine 3-beta-galactosyltransferases was found in PWSs individuals compared with all other stations, although there were some exceptions (Fig. 7).

Lipid transport involves multiple functions including the transport associated with the production of new lipid resources. Expression of genes involved in lipid transport was uniformly downregulated in CVs from the more offshore stations (Fig. 7). Two FA transporters and two fatty acid binding proteins (*FABP5* and *FABP6*) were upregulated in PWS individuals compared with individuals from the Gulf of Alaska, with *FABP6* showing also high expression in GAK1 individuals (Fig. 7). However, there was one *FABP6* gene with high expression in GAK4 and GAK9 CVs. Upregulation of eight synaptotagmins, was observed in PWS2 and GAK4 individuals. Synaptotagmins are transmembrane transporters, which are typically involved in signal transduction.

Upregulated genes in individuals from the offshore stations were predominantly involved in catabolic processes (Fig. 7). While in many cases these genes were upregulated in individuals from the two most offshore stations, expression was typically higher in GAK14 than in GAK9. In nearly all the categories, relative expression of genes involved in lipid catabolism showed the opposite pattern of the lipid synthesis genes—upregulation in offshore individuals, intermediate expression in GAK1, and low expression in PWS individuals. Transcripts encoding all enzymes involved in FA catabolism (β-oxidation), *acyl-CoA synthase*, *acyl-CoA dehydrogenase*, *enoyl-CoA hydratase*, and *3-hydroxyacyl-CoA dehydrogenase* were more highly expressed in individuals from GAK9 and GAK14 than those from all other stations (Fig. 7). Genes involved in glycolipid catabolism were upregulated in the offshore individuals and this included *glycerophosphocholine phosphodiesterase*, *glycerol-3-phosphate phosphatase* and *peroxidin 6* (Fig. 7). Among the enriched GO terms were transcripts encoding proteins involved in sphingolipid and phospholipid metabolism. Their expression pattern was variable: some catalytic enzyme-encoding genes (a single *prosaposin*, *sphingosine-1-phosphatase* and *sphingosine-1-phosphate lyase*) were upregulated in individuals from GAK9 and GAK14, while others were upregulated in those from the more inshore stations (Fig. 7).

**Fig. 8:**
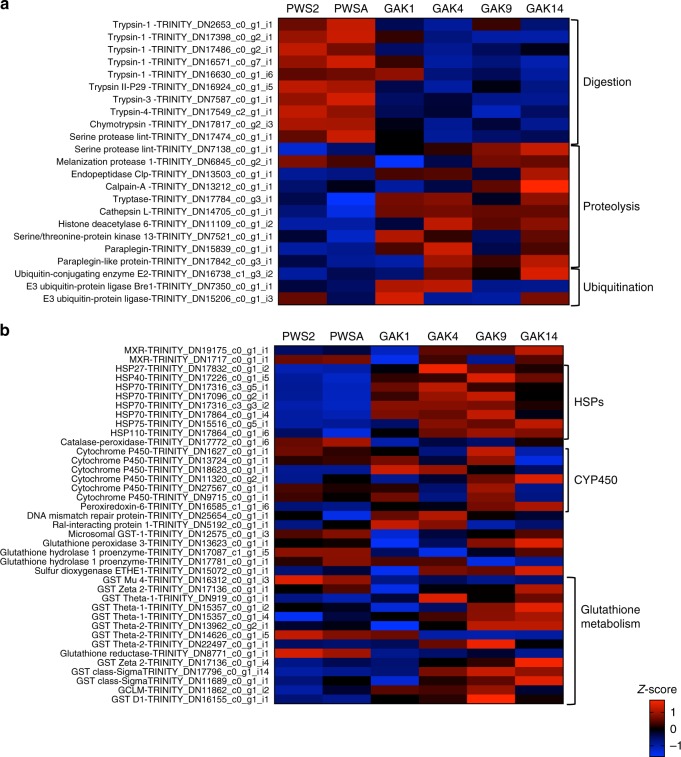
Pattern of expression of genes involved in protein metabolism, response to stress and glutathione metabolism in *Neocalanus flemingeri* CVs. Heatmap for differentially expressed genes (DEGs) involved in **a** ‘protein metabolic process’ [GO:0019538], ‘protein ubiquitination’ [GO:0016567] and **b** ‘response to stress’ [GO:0006950] and ‘glutathione metabolism’ [GO:0006950, GO:0006749]. Color coding for each gene (scale bottom right) indicates the magnitude of differential expression among stations calculated as the mean *z*-score averaged for individuals from that station (*n* = 3, except for GAK9 *n* = 2). Station identified above each column. Genes were grouped by function as indicated by the brackets on the right. Gene annotations and their Trinity ID number are listed on the left. Annotation *E*-values for this set of genes ranged from 1e−10 to 1e−150 (Supplementary data 4)

In addition to lipid catabolism, genes involved in protein catabolism were upregulated in individuals from the offshore stations. Catabolism of proteins in combination with lipid catabolism are the indicators of nutritional stress in crustaceans^28^. In *N. flemingeri* CVs, genes involved in proteolysis included several transcripts (*serine protease*, *endopeptidase*, *paraplegin*) that were highly expressed in GAK9 and GAK14 individuals. Genes coding an *endopeptidase*, *melanization protease 1*, *cathepsin* were significantly upregulated (*p* value ≤ 0.05) in GAK9, GAK14, as well as GAK4 individuals (Fig. 8a). Furthermore, DEGs involved in protein ubiquitination, an enriched GO term, were upregulated in GAK14 individuals as shown by the expression pattern of *E3 ubiquitin * and *E2* conjugating enzyme  (Fig. 8a). In contrast to this pattern, digestive enzymes (nine DEGs annotated as *trypsins* and *chymotrypsins*) were upregulated in PWS individuals (PWS2, PWSA) compared with those from the GAK stations (Fig. 8a). In marine crustaceans, downregulation of trypsins and chymotrypsins occurs under food-limited conditions^28^.

#### Response to stress

Relative expression of DEGs (*n* = 39) involved in response to stress is shown in Fig. 8b. Transcripts coding stress proteins (*hsps*, *catalase peroxidase*, *DNA mismatch*) and multixenobiotic response (*MXR*) proteins were downregulated in PWS individuals compared with those from the GAK stations (Fig. 8b). However, pairwise comparisons, identified the GO terms ‘response to stress’ and ‘glutathione metabolism’ enriched   specificailly in either GAK4 or GAK9 individuals (Supplementary data 2). A large number of these genes were upregulated in either GAK4 or GAK9 individuals or both (*n* = 31; Fig. 8b). Eight heat shock protein genes (e.g. *hsp27*, *hsp70*, *hsp110*) were highly expressed in GAK4 and GAK9 individuals. An additional three hsps (*hsp40*, *hsp75*, and another *hsp110*) were also upregulated at GAK14 (Fig. 8b). Two multixenobiotic resistance protein genes (*MXR*) were significantly high expressed in GAK4 individuals raising the possibility of a localized source of xenobiotic stress at that station. Among the DEGs five out of six *cytochrome p450* (*CYP450*) genes were upregulated in GAK9 individuals with one of them being also upregulated in GAK14 (Fig. 8b).

Glutathione metabolism genes are involved in many biological processes. While many genes are regulated in response to oxidative stress, they are also involved in nutrient metabolism, DNA and protein synthesis, signal transduction, and immune response^29^. Within glutathione metabolism (DEGs = 19), the majority of the genes were upregulated at three GAK stations (GAK4, GAK9, and GAK14) as shown by the the high expression of four *glutathione S-transferases* (*GST*: three *theta* and one *sigma*) and *a persulfide dioxygenase* (Fig. 8b). *A glutathione peroxidase* and three additional *GSTs* (one *theta 1*; one *sigma* and one *zeta*) were upregulated in individuals from the most offshore stations (GAK9 and GAK14), while a single *glutamate-cysteine ligase* (GCLM) and *a GST delta* were upregulated in GAK9 individuals. In addition, several genes were more highly expressed in PWS individuals than in those from the GAK stations. These genes included *a microsomal GST*, *a glutathione hydrolase 1 proenzyme*, *a glutathione hydrolase*, and three additional *GSTs* (*theta* 1, *theta* 2, and *mu* 4).

#### Development

**Fig. 9:**
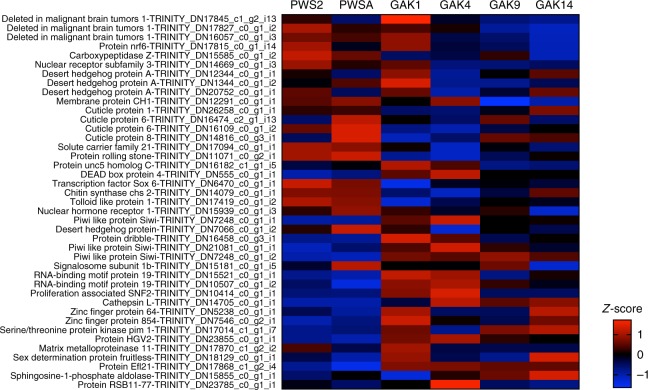
Pattern of expression of genes involved in development in *Neocalanus flemingeri* CVs. Heatmap for differentially expressed genes (DEGs) involved in ‘developmental process’ [GO:0032502]. Color-coding for each gene (scale bottom right) indicates the magnitude of differential expression among stations calculated as the mean *z*-score averaged for individuals from that station (*n* = 3, except for GAK9 *n* = 2). Station identified above each column. Genes were grouped by function as indicated by the brackets on the right. Gene annotations and their Trinity ID number are listed on the left. Annotation *E*-values for this set of genes ranged between 1e−08 and 1e−167 (Supplementary data 4)

Although the population cycle of *N. flemingeri* is highly synchronized to the seasonal cycle, there is some variability in the specific timing of the disappearance of CV stage individuals from the surface layer^21,24^. Enrichment analysis identified development as an of the over- represented GO term among the DEGs (Fig. 6). Similar to other enriched processes, genes involved in development showed regional differences in expression, as shown in the heatmap of 41 DEGs (Fig. 9, Supplementary data 2). The GO term ‘multicellular organism development’ [GO:0007275] was enriched in six pairwise comparisons with either PWS2 or PWSA against the GAK stations (Supplementary data 2). Differences in expression occurred between inshore (PWS2, PWSA), intermediate (GAK1, GAK4) and offshore (GAK9, GAK14) individuals (Fig. 9). Sixteen DEGs were upregulated in either PWS2 or PWSA stations compared with GAK stations. This included transcripts encoding three *cuticle proteins*, a single *deleted in malignant brain tumors 1 protein*, a *nuclear receptor*, a *transcription factor Sox* 6 and *tolloid-like proteins* (Fig. 9). Significant higher expression (*p* value ≤ 0.05) in individuals from GAK1 and/or GAK4 was found for several genes (e.g. *Piwi-like proteins*, a *desert hedgehog protein* and *DEAD box protein*) (Fig. 9). Some of these genes (e.g. *cathepsin*, *serine/threonine kinase)* were also upregulated in the offshore station (Fig. 9). Transcripts for two genes (*Piwi-like* and *Efl21*) were upregulated in all GAK stations compared with PWS (Fig. 9). Specific to GAK14 individuals was the upregulation of a single* cuticle* *protein* and a *zinc finger protein 64* (Fig. 9). While it is difficult to relate this differential gene expression to specific developmental processes in *N. flemingeri*, it suggests that these stage CV individuals from different stations varied in their developmental progression towards the final molt and maturation.

## Discussion

*N. flemingeri*, a species that contributes to the high-latitude marine lipidscape^23^, depends on a short and variable annual phytoplankton bloom to: (1) mature and prepare for diapause; and (2) reach a location suitable for diapause (≥400 m depth)^24^. The persistence of *N. flemingeri* in the northern Gulf of Alaska and the subarctic Pacific indicates that enough individuals meet these two challenges in spite of high advection rates, patchy resource availability and interannual variability in the timing and magnitude of the spring bloom^18^. Long-term monitoring in the northern Gulf of Alaska indicates that *N. flemingeri* abundances fluctuate year-to-year by fourfold or greater, indicating significant differences in recruitment and/or survival^25^. While the reasons for differences in abundance are numerous, variability in growth conditions is likely to be an important contributor^30^. However, projected increases in global temperatures and extreme climatic events have raised the question whether the resilience in the *N. flemingeri* population observed heretofore is long-term stable. While the current study does not address this question directly, it provides an ecophysiological framework for evaluating transcriptional signatures within the context of the copepod’s life history and its environment during diapause preparation.

Steep environmental inshore–offshore gradients in nutrient availability, primary production, and phytoplankton standing stocks characterize the Gulf of Alaska^31^. Gradients in the size distribution of autotrophs are typical with larger phytoplankton cells and higher abundances consistently occurring in PWS and nearshore areas compared with mid- and outer-shelf areas^30^. In addition, coastal upwelling and downwelling, nearshore circulation patterns, and large-scale physical features like mesoscale eddies and meanders add complexity and unpredictability to advection patterns and food availability^18,19,31–34^. Thus, during the March to May growth period, any individual *N. flemingeri* can be advected by 100 km or more and may experience a patchy food environment that varies stochastically and is rarely optimal^19^. Furthermore, advection leads to basin-scale mixing of zooplankton populations preventing local genetic adaptation^19,35^. We observed no regional differences in genotypes using mitochondrial and nuclear markers consistent with basin-scale connectivity across the region. In contrast, physiological acclimatization to environmental conditions is suggested by large regional differences in gene expression. Observed differences were indicative of both short term (hours to days: response to stress, metabolism) and more long-term (days to weeks: digestive enzymes, development) responses to environmental conditions.

In early May 2015, food resources declined from inshore to offshore as would be expected for the northern Gulf of Alaska^19,35^. Even though *N. flemingeri* feeds on heterotrophs, and even prefers large ciliates^36^, large phytoplankton cells remain the dominant dietary component during May^36–38^. *Neocalanus flemingeri *feeds selectively on large cells (>20 µm), and chlorophyll *a* concentrations in this size fraction differed by more than an order of magnitude between the offshore stations (GAK9 and GAK14) and Prince William Sound (integrated chl *a* from 2 to >100 mg m^−2^). Given chlorophyll *a* concentrations at the six stations in May 2015 and extrapolating from experimental measurements of ingestion rates by Dagg et al.^37^, we estimate that *N. flemingeri* ingestion rates could have ranged from ~20 (GAK9 and GAK14) to ~250 (PWSA) ng chl*a* per copepod per day. Thus, local environmental conditions were near optimal in Prince William Sound and increasingly resource-limited from inshore to offshore along the Seward Line.

Diapause preparation in arthropods includes the buildup of lipid stores prior to developmental arrest^39^. While lipid accumulation starts in the early to mid copepodid stages in *N. flemingeri*^40^, it is particularly prevalent during the pre-adult stage in all diapausing calanid copepods^41–43^. Because *N. flemingeri* does not feed as an adult, lipid stores accumulated during development in April and May fuel both diapause and the recruitment of the next generation^24^. Inshore–offshore gradients in productivity are strong in the Gulf of Alaska: broad-scale patterns of productivity co-occur with finer-scale heterogeneity as seen in satellite images^44^. Biophysical simulation models that combine a circulation model (ROMS) with a lower trophic level component (NPZ model) suggest that observed gradients in resources (i.e., nutrient limitation, chlorophyll *a*) can persist over time^44–46^. Thus, individual *N. flemingeri* can experience conditions that are suboptimal for growth and the accumulation of lipid stores for extended time periods, which in turn would impact postdiapause reproductive success^32^. Furthermore, the current study occurred during the marine heat wave of 2014–2016^45–47^, which may have created additional nutritional stress on zooplankton, as well as other marine species^47^.

Differences in the expression of genes associated with development across stations might be indicative of differences in the progression towards the final molt into the adult stage. While developmental rates are affected by temperature, temperature differences across the study site were modest. However, differences in resource availability (i.e chlorophyll *a*) can affect developmental rates by as much as a factor of two^48^. Furthermore, phytoplankton blooms typically occur earlier in PWS than in the Gulf of Alaska^44^. As with most copepods, ingestion rates in *N. flemingeri* vary directly with chlorophyll *a* concentration, and are highest above 1.5 mg m^−3^ ^37^, concentrations that were present in the large-cell fraction (>20 µm) in PWS, but not in the Gulf of Alaska (GAK1-14).

Surface salinity at GAK14 and a predominance of chlorophyll *a* in the small cell fraction suggests it is very close to the HNLC waters characteristic of the central Gulf of Alaska^21,32^. The effect of nonoptimal food conditions on zooplankton has been difficult to study in the natural habitat given the dynamic nature of the pelagic environment^18^. Based on grazing studies, ingestion rates of *N. flemingeri* would have been high at the concentrations of large-cell  chlorophyll *a* observed in PWS, intermediate at GAK1, and low at GAK4-14. To our knowledge, our study is the first to correlate an inshore-offshore gradient in the abundance of large  phytoplankton cells to large differences in gene expression in a zooplankter. Signs of increased nutritional stress along the Seward Line (GAK1 to GAK14) in the Gulf of Alaska are suggested by differences in expression of genes involved in lipid and protein metabolism. In mosquitoes, lipid accumulation during diapause preparation is characterized by the upregulation of genes involved in FA synthesis, consistent with high expression of these genes in PWS individuals. Expression of these genes was increasingly down-regulated in a nearshore–offshore pattern in individuals collected along the Seward Line. Furthermore, we found simultaneous upregulation of genes involved in the β-oxidation pathway in individuals from three Seward Line stations (GAK4, GAK9, and GAK14): a pattern that is indicative of current metabolic needs being fueled by fat breakdown, which occurs in organisms under nutritional deprivation^49,50^. Even greater nutritional deficits at GAK9 and GAK14 are suggested by upregulation of genes involved in proteolysis and downregulation of digestive enzymes, which are related to low food availability^28^. Furthermore, in the most offshore station (GAK14), genes involved in the ubiquination system were upregulated, a pattern associated with food deprivation in mammals^51^.

Individuals from two stations in the Gulf of Alaska (GAK4 and GAK9) showed evidence for the activation of a cellular stress response, which protects organisms from environmental stressors^52^. These two intermediate stations are located in a transition zone between the Alaska Coastal Current and the Alaska Stream, a region that is characterized by complex hydrography^21,31,35,44^. Overall more than 8% of the DEGs were annotated with the GO term ‘response to stress’, and 40% of these were among the upregulated DEGs in GAK4 and GAK9 individuals only. A generalized response, characterized by the upregulation of several heat shock proteins, CYP450s, and GST, was observed at both stations. In addition, GAK4 individuals showed upregulation of detoxification genes associated with the MXR system, which is activated in response to environmental toxins^52^. While the specific stressors are currently unknown, the gene expression patterns suggest two different sources of stress at two stations that are ~90 km apart. Fine-scale environmental heterogeneity in this region could lead to the presence of localized chemical and/or biological stressors^20,21,25,31,32,35,44^.

Transcriptional profiling demonstrated large differences in gene expression among individuals along an inshore–offshore gradient from Prince William Sound to the outer shelf in the Gulf of Alaska (GAK14), suggesting physiological acclimatization to local conditions. Upregulation of lipid synthesis genes in PWS individuals is consistent with accumulation of lipids associated with diapause preparation. In contrast, CV individuals from GAK9 and GAK14 did not appear to be building lipid stores given the up-regulation of genes involved in lipid catabolism and protein degradation. Nevertheless, *N. flemingeri* persists in this region in spite of low food conditions, evidence of species resiliency. An ability to exploit even brief pulses of high food to build lipids to fuel diapause and reproduction, while acclimatizing to low food conditions, may contribute to the resilience of this species. The transcriptomic evidence suggests that food conditions in May 2015, were optimal in PWS, but they may not have been sufficient to fuel diapause and reproduction in the offshore GAK individuals in the absence of an injection of new resources later in the season.

While the results indicate physiological acclimatization in *N. flemingeri*^53^, in future studies the observed transcriptional signatures need to be linked to lipid accumulation rates and development under experimental conditions. Experimental calibration of relative expression patterns would provide a basis to quantify how spatial and interannual environmental variability affects the physiology of *N. flemingeri* during the growth period and diapause, and reproductive success. With diapausing females consistently found at depth in western Prince William Sound^54^, there is an opportunity to compare *N. flemingeri* population assessments of pre-adults in May, diapausing adults in July–September and nauplii in the following spring (March) with gene expression profiles and in situ growth rates. Such studies would add to an understanding of how acclimatization in *N. flemingeri* contributes to its resilience.

## Methods

### Sampling strategy and environmental data

In collaboration with the Seward Long-Term Observation Program (http://www.sfos.uaf.edu/sewardline/), we obtained *N. flemingeri* CV individuals during the annual May oceanographic cruise from six locations: four stations spanning the inner shelf to outer shelf gradient along the Seward Line in the northern Gulf of Alaska and two stations in adjoining Prince William Sound (Fig. 1b). Samples were collected between May 5 and 10, 2015 using a CalVET net (53-µm mesh) towed vertically from 100 m depth to surface. Mixed plankton samples were immediately diluted with surface seawater, and maintained at ~5 °C prior to and during sorting. From each station actively swimming (healthy) *N. flemingeri* CVs were rapidly sorted under the microscope and preserved within 2 h of the tow in RNAlater Stabilization Reagent (QIAGEN). Temperature and salinity were measured using SBE 911 + CTD at all stations to the bottom or a maximum depth of 1000 m. The CTD was connected to a SBE32C rosette with 16 Niskin water-sampling bottles used to collect water for chlorophyll *a* in the upper 50 m at a 10-m interval. Water samples were filtered serially through 20 µm Poretics polycarbonate filters and onto Whatman GF/F filters under dim light at low pressure. Chlorophyll was then extracted immediately at −20 °C in 90% acetone for the two size fractions (<20 µm and >20 µm) and read fluorometrically after 24 h. Collection information dates, times, and locations are provided in Table 1.

### RNA extraction, gene library preparation and RNA-Seq

Total RNA was extracted from individual CV from each station using the QIAGEN RNeasy Plus Mini Kit (catalog # 74134) in combination with a QIAshredder column (catalog # 79654) following the instructions of the manufacturer and stored at −80 °C. Total RNA concentration and quality were checked using an Agilent Model 2100 Bioanalyzer (Agilent Technologies, Inc., Santa Clara, CA, USA). For each station, total RNA from three of the ten individuals with high quality RNA yields were selected for RNA-Seq and shipped on dry ice to the University of Georgia Genomics Facility (dna.uga.edu). There double-stranded cDNA libraries were prepared from total RNA extracted using the Kapa Stranded mRNA-seq kit (KK8420) following manufacturer’s instructions. Briefly, RNA samples were first purified with two oligo-dT selection (polyA enrichment using oligo-dT beds), and then fragmented and reverse transcribed into double-stranded complementary DNA. Each sample was tagged with an indexed adapter and they were simultaneously paired-end sequenced (PE150 bp) using an Illumina NextSeq 500 instrument using a High-Output Flow Cell. The quality of each RNA-Seq library (*n* = 18) was assessed using FASTQC (v1.0.0; Illumina Basespace Labs). The first 9 bp and any remaining Illumina adapters (TruSeqLT universal primer) were trimmed from each read using FASTQ Toolkit (v.2.0.0; Illumina Basespace Labs). This was followed by the removal of reads with low average quality (Phred score < 30), which led to the removal of an average of 8% reads from each library. After the initial filtering, reads were checked for matched pairs, which resulted in an additional removal of 20–30% from each library. Quality filtering of each library resulted in 7–15 million reads per sample with an average of 9 million (Supplementary Table 1).

### Development of individual de novo assemblies

Separate de novo assemblies were generated for each of the 18 individuals using Trinity software (v. 2.0.6) as described in the Supplementary Methods. De novo assemblies were searched for comparable sequences of genetic marker genes to calculate genetic distances among individuals from different stations. Based on the previous population genetics studies of *N. flemingeri*^55^, we targeted the mitochondrial genes 12S, 16S, COI, and the nuclear ribosomal 18S gene. Each assembly was mined for the target genes using BLAST software (*blastn*) installed on an Intel-processor-based BEOWULF computer cluster (Pacific Biosciences Research Center, University of Hawai’i at Mānoa, Honolulu, HI, USA). The resulting transcripts from each individual were separately aligned with their query using MAFFT (v.7.305b)^56^ and manually edited to match the length of the query. The program Arlequin (v.3.5.2.2)^57^ was used to compute analysis of molecular variance (AMOVA) and to calculate pairwise *F*_ST_ values between stations based on genetic divergence and their significance (1000 permutations). The analysis was run independently for the nuclear ribosomal 18S gene and the three concatenated mitochondrial genes (12S, 16S, COI; Mesquite, v.3.51)^58^. Cladogram based on the mtCOI sequences was generated using the identified mtCOI sequences for each *N. flemingeri* individual (PWS2 to GAK14), a reference *N. flemingeri* mtCOI sequence ^55^ and a reference mtCOI sequence from *Labidocera sp*. as the outgroup (NCBI Accession number: KC594129)^59^. RAxML (Galaxy version 1.0.0) was used to construct a maximum-likelihood tree with 1000 fast bootstraps on the best likelihood tree constructed with the GRT + gamma model.

Because Trinity software retains existing isoform diversity, combining RNA-Seq reads from multiple individuals that originated from a large population like *N. flemingeri* leads to highly fragmented assemblies^60^. This fragmentation is not easily corrected, even after assembling sequences using the CAP3 program^60^. In contrast, de novo assemblies obtained from reads obtained from single individuals are of high quality based on overall mapping, sequence length and BUSCO^61^ analysis (Supplementary data 3). After comparing across the 18 assemblies, the de novo assembly from a GAK1 individual (GAK1-S83R1, sequencing depth: 1.9Gb; NCBI Accession number: SRX4908946) was selected as the reference transcriptome for the short-read mapping step. A summary of assembly and annotation statistics is shown in Supplementary Table 3. Mapping bias was checked by mapping quality-filtered reads from all of the libraries (*n* = 18) back to this reference transcriptome (Supplementary Methods) using Bowtie2 software (v2.1.0)^62^ (Supplementary data 3). Except for one individual (GAK9-S18-S7; 82%), mapping statistics were similar across samples and ranged between 89 and 95% for all samples (Supplementary Fig. 1 and Supplementary data 3). The mapping step was followed by an agnostic comparison across gene expression profiles. A hierarchical clustering approach was used to identify similarity/dissimilarity of gene expression patterns among individuals independent of their collection site. After the mapping step using Bowtie2, normalized relative expression was calculated from the counts by dividing each count by the number of mapped reads (per million) and by the length of the gene (per kilobase) using the RPKM method^63^ as implemented by edgeR without the TMM normalization step^64^. Individuals (*n* = 18) were then clustered using the function *hclust* (R package, v 3.6.0) using the average linkage method (UPGMA), and all other default settings^65^. The individual with the 82% mapping rate (GAK9-S18-S7) clustered separately from the other individuals in the genetic cladogram (mtCOI gene). Due to this uncertainity, we did not include this GAK9 individual in the remaining downstream analyses.

### Identification of differentially expressed genes among stations

DEGs among stations were identified using a Generalized Linear Model (GLM) with subsequent pairwisecomparisons (glmLRT) between stations.  Three individuals were considered per station, with the exception of GAK9 (two individuals). Following the workflow optimization recommended by Trinity (https://github.com/trinityrnaseq/trinityrnaseq/wiki/Trinity-Transcript-Quantification) statistical analysis was performed on reads mapped to the reference transcriptome using kallisto software (default settings; v.0.43.1) to reduce potential errors associated with ambiguous mapping^66,67^. After mapping, RNA-Seq libraries with relative transcript abundances were analyzed for differential gene expression analysis using the BioConductor package edgeR (R package; v. 3.24.3)^64^. As implemented by edgeR, the RNA-Seq libraries were normalized using the TMM methods (trimmed means of M values) prior to statistical testing. This step was followed by the removal of transcripts with expression levels below 1 count per million (1 cpm). Statistical testing for differential gene expression on the remaining 47021 transcripts was performed across stations using the negative binomial generalized linear model (glmFit). *P*-values were adjusted with the Benjamini–Hochberg procedure to control for false discovery rate (FDR). A gene was considered differentially expressed if its adjusted *p* value ≤ 0.05. Significant differences in gene expression between station pairs were determined using the downstream pairwise likelihood ratio tests (glmLRT) (*p* value ≤ 0.05) using the BioConductor package edgeR^64^.

Functional analysis of DEGs was performed by searching the DEGs from the GLM analysis against the annotated reference transcriptome (24356 transcripts) which resulted in 3107 DEGs with GO terms. Enrichment analysis was performed on the DEGs from the pairwise comparisons (glmLRT) which retrieved GO terms. These DEGs were combined and compared against the 24356 transcripts with GO terms in the reference transcriptome using TopGO software (R package; v. 2.88.0)^68^. The analysis was performed using the default algorithm weight01 employing the Fisher exact test and a Benjamini–Hochberg correction with a *p* value < 0.05 to obtain lists of enriched GO terms for each pairwise comparison (*n* = 15; Supplementary data 2). The software reviGO was used to summarize and visualize these enrichment results. As implemented by reviGO, a redundancy reduction is applied to the GO term list and the resulting GO terms and their pvalues are then visualized in a in a two dimensional space derived by applying multidimensional scaling to a matrix of the GO terms’ semantic similarities^69^. ReviGO's guiding principle is that semantically similar GO terms should remain close together in the plot. Bubble color indicates the user-provided *p* value and size indicates the frequency of the GO term in the Gene Ontology database (bubbles of more general terms are larger). For the analysis, the list of enriched GO terms (from each pairwise comparison) with their pvalues and FDR was uploaded in reviGO (http://revigo.irb.hr) using a similarity setting to medium (0.7).

Based on the enrichment analysis heatmaps for DEGs involved in metabolism (lipid and protein), response to stress and development were generated. AMIGO software GOOSE (April, 2019)^70^ was used to search the list of annotated DEGs (*n* = 3107) for the following GO terms (‘lipid metabolic process’ [GO:0006629], ‘lipid transport’ [GO:0006869], ‘protein metabolic process’ [GO:0019538], ‘protein ubiquitination’ [GO:0016567], ‘protein digestion’ [GO:0044256], ‘response to stress’ [GO:0006950], ‘glutathione metabolic process’ [GO:0006749], ‘developmental process’ [GO:0032502]), and its descendants using the LEAD SQL wiki called “find descendants of the node ‘nucleus’ with ‘nucleus’ replaced with the specific GO term name (e.g. ‘lipid metabolic process’). Relative expression for the DEGs is shown as the *z*-score computed for each individual or averaged across station using log(RPKM) calculated from the counts generated by kallisto software^66,67^, with RPKM normalized as implemented by edgeR including the TMM normalization step^64^.

### Statistics and reproducibility

Analysis for genetic variation was performed using Arlequin (v.3.5.2.2)^57^. AMOVA was run independently on the four target genes using a total of 18 individuals (three individuals per station) to calculate pairwise *F*_ST_ values between stations (1000 permutations, *p* < 0.05). Gene expression analysis was implemented in RStudio using the BioConductor package edgeR (R package; v. 3.24.3)^64^. For statistical comparison (three individuals per station with the exception of GAK9 with two individuals) the negative binomial Generalized Linear Model (glmFit function) was used and -p values (*p* value ≤ 0.05) were adjusted with the Benjamini–Hochberg procedure to control for FDR. Downstream pairwise comparisons using likelihood ratio test (glmLRT) with *p* value ≤ 0.05 was impemented to identify station-specific differences. Fisher’s exact test was adopted to compute gene ontology enrichment with pvalues corrected using Benjamini–Hochberg methods using TopGO software (R package; v. 2.88.0)^68^.

### Reporting summary

Further information on research design is available in the Nature Research Reporting Summary linked to this article.

## Supplementary information

Supplementary Information

Description of Additional Supplementary Files

Supplementary data 1-3

Supplementary data 4

Reporting Summary

## Data Availability

The RNA-Seq datasets generated during the current study are available as raw short sequence read data for all the libraries (*n* = 18) (NCBI; BioProject: PRJNA496596). The shotgun assembly used as reference transcriptome in the paper is available at DDBJ/EMBL/GenBank under the accession GHLB01000000. The version described in this paper is the first version, GHLB01000000 (NCBI; BioProject: PRJNA496596). The data used for downstream analsyis (e.g. list of differentially expressed genes with their relative expression as RPKM) is included in the Supplementary data 4. Environmental data are available through the Alaska Ocean Observing System [https://portal.aoos.org/old/gulf-of-alaska#metadata/e25fe1f2-1c98-44f6-856f5d61c87c0384/project/folder_metadata/24099].
