# Supplementary material for: Regional heterogeneity impacts gene expression in the subarctic zooplankter Neocalanus flemingeri in the northern Gulf of Alaska
Source: Commun Biol. 2019 Sep 2;2:324. doi: 10.1038/s42003-019-0565-5 (PMC6718390; doi:10.1038/s42003-019-0565-5)
Supplement: Supplementary file 1 — Supplementary Information [file 42003_2019_565_MOESM1_ESM.pdf]

Supplementary Figures

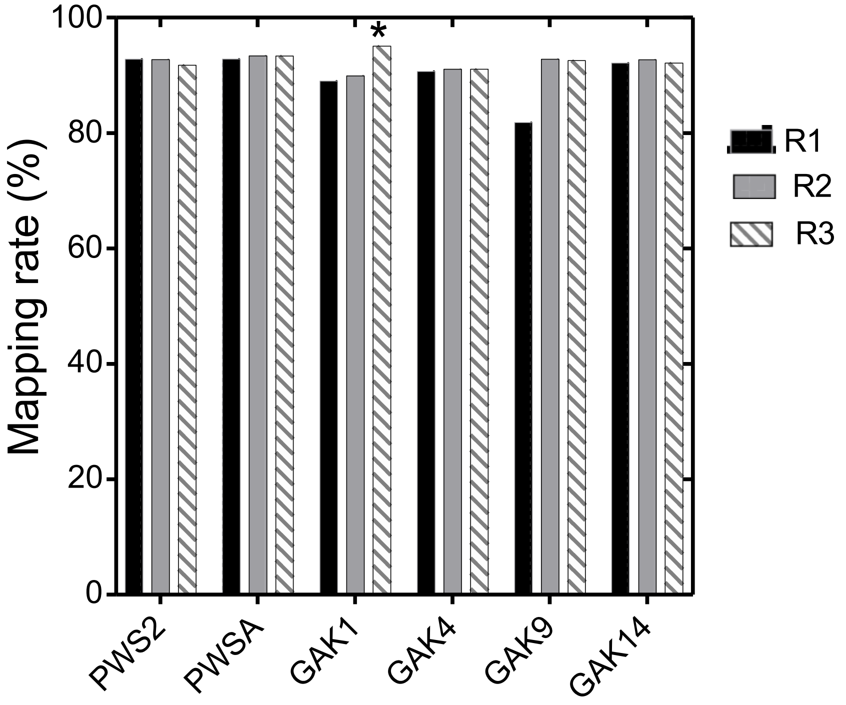

Fig. 1. Mapping rate against reference transcriptome. Alignment rate (%) of RNA-Seq reads mapped to the *de novo* reference transcriptome assembled from the reads of a single individual from GAK1 (R3 in graph- GAK1- S83). Mapping rate of three individuals (R1, R2, R3) are shown for each station as indicated in legend. Individuals follow the order of samples in Supplementary Table 1. The individual with a mapping rate of 82% (GAK9, R1) was removed from downstream analyses.

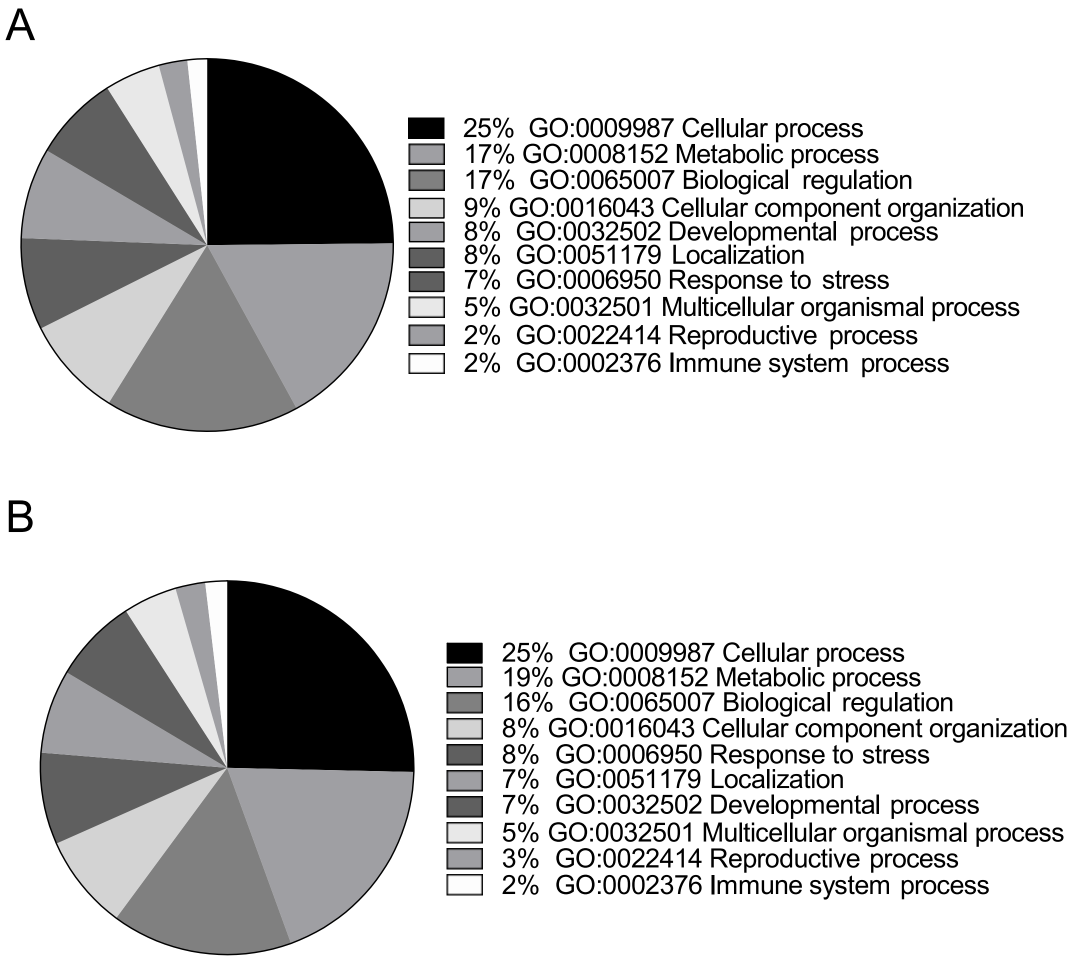

Fig. 2. **Distribution of gene ontology (GO) terms.** Pie charts showing GO annotation for: A) reference transcriptome (GAK1- S83), and B) annotated differentially expressed genes (n=3,107) (GLM, $p \leq 0.05$ after FDR correction). In both graphs percentages are shown for GO terms within different categories in biological process (BP). Numbers of transcripts annotated in each GO term: A) 'cellular process' = 8,143, 'metabolic process' = 5,626, 'biological regulation' = 5,520, 'cellular component organization' = 2,857, 'localization' = 2,622, 'developmental process' = 2,583, 'response to stress' = 2,457,'multicellular organismal process' = 1,579, 'reproductive process' = 819,'immune system process' = 557; B) 'cellular process' = 1,448, 'metabolic process' = 1,094, 'biological regulation'= 860, 'cellular component organization' = 461, 'response to stress' = 448, 'localization' = 427, 'developmental process' = 405, 'multicellular organismal process' = 264, 'reproductive process' = 152, 'immune system process' = 105].

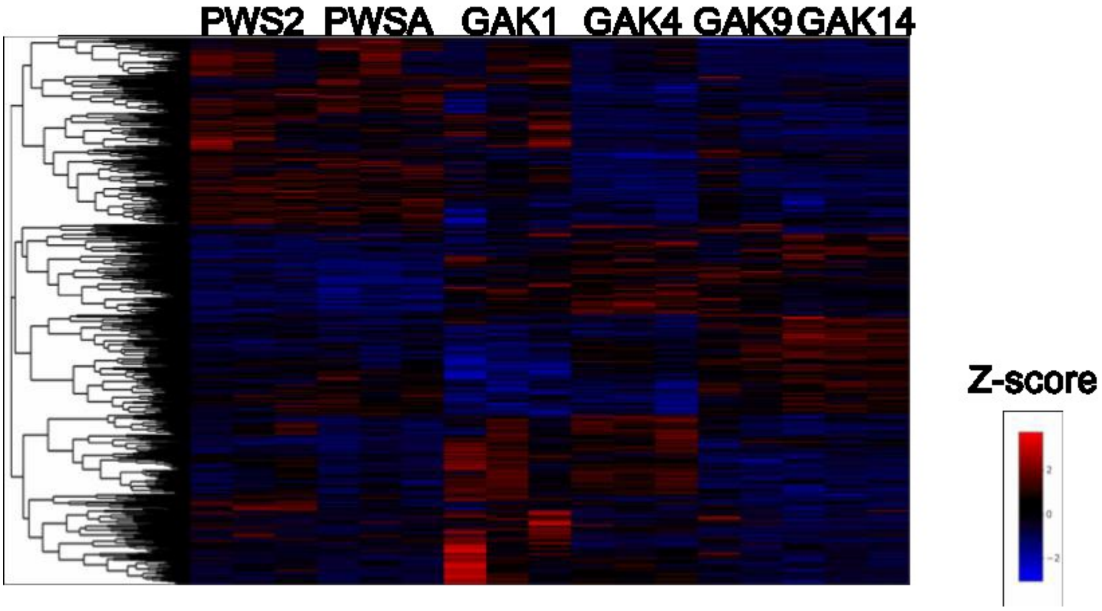

Fig. 3. **Relative gene expression in in individual *Neocalanus flemingeri* CVs.** Heatmap of z-scores of differentially expressed genes (DEGs, rows) that did not annotate at an E-value 1e-05 or lower (n=3,365). Color-coding for each gene indicates the magnitude of differential expression calculated as the z-score (scale bottom right). Each column represents relative expression of an individual with collection station identified above. All stations include three individuals with the exception of GAK9 (see Fig. 4B, Supplementary Figure 1). Genes were ordered by similarity of expression pattern as shown by the dendrogram (left). DEGs were identified by GLM test with $p \leq$ 1.5 after FDR correction.

Supplementary Tables

Supplementary Table 1. Summary of RNA-Seq results for *Neocalanus flemingeri* CV individuals collected at sample locations within Prince William Sound and Gulf of Alaska. RNA-Seq was performed on pre-adults (copepodite stage CV) collected from six stations (three individuals per station) within Prince William Sound (PWS) and Gulf of Alaska (GAK). For each individual, number of RNA-Seq reads, highly-quality reads and NCBI accession numbers are provided. Raw reads have been deposited at National Center for Biotechnology Information (NCBI) under Bioproject No. PRJNA496596.

**Stations**	Sample ID	Accession #	RNA-Seq reads (PE)	High quality PE reads
PWS2	PWS2-S54	SRX4908800	14,835,282	9,536,693
	PWS2-S53-R2	SRX4908801	15,162,793	9,903,603
	PWS2-S55-R1	SRX4908802	15,916,270	10,202,038
PWSA	PWSA-S60-R4	SRX4908804	12,361,268	7,773,905
	PWSA- S60-R1	SRX4908805	14,526,721	9,279,570
	PWSA-S60-R2	SRX4908806	12,824,228	8,412,977
GAK1	GAK1-S80-R3	SRX4908944	13,306,923	8,973,744
	GAK1-S82	SRX4908945	15,574,562	10,264,314
	GAK1-S83-R1	SRX4908946	17,198,201	11,389,456
GAK4	GAK4-S94-R3	SRX4915135	17,686,832	11,705,826
	GAK4-S94-R1	SRX4915136	15,211,747	10,200,000
	GAK4-S94-R2	SRX4915137	13,712,810	9,240,995
GAK9	GAK9-S18	SRX4915140	14,492,063	9,455,782
	GAK9-S20-R3	SRX4915141	15,162,793	9,903,603
	GAK9-S20	SRX4915142	15,338,923	10,170,204
GAK14	GAK14-S16-R3	SRX4915172	14,235,936	8,876,985
	GAK14-S15	SRX4915173	12,615,067	8,073,250
	GAK14-S16-R2	SRX4915174	14,107,424	9,067,277

Supplementary Table 2. Summary of the AMOVA results partitioning genetic variation among individuals collected from six stations in Prince William Sound (PWS2, PWSA) and in the Gulf of Alaska (GAK1, GAK4, GAK9, GAK14) using mitochondrial (12S, 16S COI) and the nuclear ribosomal 18S markers; df = degrees of freedom, ss = sum of squared observation, %V = percent of total variance, F_{ST} = F_{ST} values.

Marker	Source of variation	df	ss	% V	F_{ST}
Mitochondrial	Among populations	5	86.278	18.66	
	Within populations	12	122.667	81.34	
					0.18656
Nuclear	Among populations	5	57.778	-2.22	
	Within populations	12	148.333	102.2	
					-0.02221

Supplementary Table 3. **GAK1 reference transcriptome.** Summary of *de novo* assembly and annotation statistics for the transcriptome used as reference in the gene expression study.

***De novo* assembly** Trinity	Total trinity transcripts (#)	51,743
	Total trinity 'genes' (#)	32,946
	N50(bp)	1,085
**Annotation**		
Transdecoder	Transcripts with coding regions (CDS) (#)	44,621
SwissProt	Transcripts with BLAST hits (#)	24,989
Gene Ontology	Transcripts with GO terms (#)	24,356
KEGG	Transcripts with KEGG terms (#)	9,579

Supplementary Methods

Development of* de novo *assemblies and of a reference transcriptome

A separate assembly was generated for each of the 18 individuals using Trinity software (v. 2.0.6) on the National Center for Genome Analysis Support's (NCGAS; Indiana University, Bloomington, IN, USA) Mason Linux cluster. The initial parameters of Trinity were set to: --seqType fq --CPU 32 --max_memory 200G --min_contig_length 300 -- normalize_max_read_cov 50. The minimum sequence length in the assembly was set to 300 bp. For each assembly, summary of the statistics was obtained using the script TrinityStats.pl (v2.0.6). Each assembly was tested for completeness using "Bench-marking universal single-copy orthologs" (BUSCO) software (v1.22) to identify "core genes", single copy genes highly conserved among eukaryotes, and thus expected to be present in a complete assembly. BUSCO analysis was performed using the Arthropoda dataset consisting of 2,675 single-copy orthologs. These individual assemblies were used in the genetics analysis using genetic markers (three mitochondrial and one nuclear marker).

The d*e novo* assembly from a GAK1 individual (GAK1-S83R1) was selected as the reference transcriptome to identify differentially expressed genes (DEGs). Annotation of transcripts and functional analysis were performed in different steps. First, Transdecoder (v. v5.5.0)[1] (setting: report only the single best ORF) was used to predict transcripts with coding regions (cds). Then, all transcripts with predicted coding region (cds) were annotated using a local BLAST webserver on a Beowulf cluster running the NCBI BLAST algorithm[2]. Transcripts were annotated against the NCBI SwissProt protein database (blastx 2.8.1+, downloaded March 12, 2019**)** using the following settings: 'maximum E-value 1e-05' and 'maximum 5 blast hits/transcript'. Transcripts with blast annotations were then searched against the Gene Ontology (GO) and the Kyoto Encyclopedia of Genes and Genomes (KEGG) pathway database using UniProt (http://www.uniprot.org/help/uniprotkb). The annotated GAK1 transcriptome

(GAK1-S83-R1) was used as reference transcriptome for the mapping step of short sequence reads. Briefly, quality-filtered reads from all of the short-sequence-read libraries (n=18) were mapped against the reference transcriptome using Bowtie2 software (v2.1.0) in order to obtain mapping statistics for each individual against the reference. For the statistical analysis for differential gene expression, we used the software kallisto to minimize the effect of ambiguous mapping [66]. The annotated *de novo* reference transcriptome is available on the NCBI Bioproject No. PRJNA496596.

Supplementary Results

The assembly statistics for the 18 *de novo* assemblies were comparable with similar number of transcripts and N50 lengths (Supplementary data 4). In all assemblies, the longest transcript exceeded 20,000 bp with the exception of two individual (GAK9 and GAK14; Supplementary data 4). BUSCO analysis identified approximately 40% complete orthologs in each assembly. On average, 10-12% of these genes present as more than one copy (duplicated) and an additional 12-16% of core genes were fragmented in each assembly (Supplementary data 4). The number of missing core genes ranged from 25% to 45% (Supplementary data 4).

The *de novo* assembly from the GAK1 individual (S83-R1) was used as the reference transcriptome for the gene expression study. The assembly generated 51,743 transcripts with an average length of 864 bp, a maximum of 24,750 bp and an N50 value of 1,085 bp (Supplementary Table 2, Supplementary data 4). It contained 32,946 Trinity predicted genes that is transcripts with unique identifiers (Supplementary Table 2, Supplementary data 4). Of the "Trinity predicted genes", the majority (20,999) were singletons (64%), with the remaining genes (26,579) possessing from two to 29 isoforms. Transdecoder identified 44,621 transcripts with coding regions (cds) of which 56% retrieved significant hits (E-value 1e-05 or better) when blasted against SwissProt database (Supplementary Table 2). Of these annotated transcripts, 97% were

further annotated with gene ontology terms (GO) and 38% against the KEGG database (Supplementary Table 2). Within the biological process category (BP), transcripts covered broadly conserved eukaryotic processes with ’cellular process’ [GO:0009987], ‘metabolic process‘ [GO:0008152], and ‘biological regulation‘ [GO:0065007] being the most highly represented terms among the annotated transcripts (Supplementary Fig. 2A). The majority (60%) of the transcripts with KEGG annotations were involved in metabolic pathways including amino-acid biosynthesis, lipid and carbohydrate metabolism, and nucleotide metabolism. Mapping rate for the 18 RNA-Seq libraries to the GAK1 reference transcriptome was high and ranged between 82% and 95% with an average of 91.5% (Supplementary Fig. 1). Ambiguous mapping which was ~58% (reads that aligned > 1 time), is likely to be a result of the large number of multiple isoforms assembled by Trinity.

Supplementary references

1. Haas, B. J., Papanicolaou, A., Yassour, M., Grabherr, M., Blood, P. D., Bowden, J., ... & MacManes, M. D. *De novo* transcript sequence reconstruction from RNA-seq using the Trinity platform for reference generation and analysis. *Nature protocols*, **8**, 1494 (2013).
2. Altschul, S. F., Madden, T. L., Schäffer, A. A., Zhang, J., Zhang, Z., Miller, W., & Lipman, D. J. Gapped BLAST and PSI-BLAST: a new generation of protein database search programs. *Nucleic acids Res.* **25,** 3389-3402 (1997).
