# Supplementary material for: Regional heterogeneity impacts gene expression in the subarctic zooplankter Neocalanus flemingeri in the northern Gulf of Alaska
Source: Commun Biol. 2019 Sep 2;2:324. doi: 10.1038/s42003-019-0565-5 (PMC6718390; doi:10.1038/s42003-019-0565-5)
Supplement: Supplementary file 2 — Description of Additional Supplementary Files [file 42003_2019_565_MOESM2_ESM.docx]

**Supplementary data 1.** **Differential gene expression in *Neocalanus flemingeri*.** Number of differentially expressed genes between CVs collected across six stations (PWS2, PWSA, GAK1, GAK4, GAK9, GAK14) identified by GLM test with *p* ≤ 0.05 after FDR correction followed by downstream likelihood test (likelihood test, 15 paired comparisons). For each comparison total number of DEGs, up-regualted and down-regulated are provided.

**Supplementary data 2**. **Enrichment results for *Neocalanus flemingeri* differentially expressed genes.** DEGs annotated with GO terms identified in each pairwise comparisons between all stations (likelihood test, 15 paired comparisons, Supplementary Table 3) were independently enriched against the 24,356 annotated transcripts in the GAK1 reference transcriptome using TopGO (*p* value < 0.05). In each comparison, enriched GO term with its *p* value corrected with FDR is provided.

**Supplementary data 3.** ***De novo* assembly for 18 *Neocalanus flemingeri* individuals.** RNA-Seq data from copepodite stage CV were individually assembled using Trinity software. Total number of assembled reads, Trinity transcripts, Trinity “genes”, N50, N25 and N75 lengths (bp) and maximum transcript length are listed. Results of BUSCO analysis as % of core genes (complete, duplicated, fragmented and missing) are also listed. In addition, summary statistics of mapping (Bowtie2) against the reference transcriptome (GAK1-S83-R1) are provided as overall mapping rate (%) and mapping rate >1 (%).

**Supplementary data 4.** **Relative expression of *Neocalanus flemingeri* differentially expressed genes across stations.** List of the total number of DEGs (n = 6,472) identified by GLM test with p ≤ 0.05 after FDR correction shown as relative expression (RPKM) for each individual sample (n = 18). For the DEGs which retrieved annotation results from the SwissProt blast (n = 3,107), E value, SP accession No., Entry name, Protein name, EC number and GO terms are listed.
