# Supplementary material for: Regional heterogeneity impacts gene expression in the subarctic zooplankter Neocalanus flemingeri in the northern Gulf of Alaska
Source: Commun Biol. 2019 Sep 2;2:324. doi: 10.1038/s42003-019-0565-5 (PMC6718390; doi:10.1038/s42003-019-0565-5)
Supplement: Supplementary file 5 — Reporting Summary [file 42003_2019_565_MOESM5_ESM.pdf]

nature research

Corresponding author(s): Vittoria Roncalli

Last updated by author(s): Jul 2, 2019

Reporting Summary

Nature Research wishes to improve the reproducibility of the work that we publish. This form provides structure for consistency and transparency in reporting. For further information on Nature Research policies, see Authors & Referees and the Editorial Policy Checklist.

Statistics

For all statistical analyses, confirm that the following items are present in the figure legend, table legend, main text, or Methods section.

n/a	Confirmed	
☐	☒	The exact sample size (*n*) for each experimental group/condition, given as a discrete number and unit of measurement
☐	☒	A statement on whether measurements were taken from distinct samples or whether the same sample was measured repeatedly
☐	☒	The statistical test(s) used AND whether they are one- or two-sided<br>*Only common tests should be described solely by name; describe more complex techniques in the Methods section.*
☒	☐	A description of all covariates tested
☐	☒	A description of any assumptions or corrections, such as tests of normality and adjustment for multiple comparisons
☐	☒	A full description of the statistical parameters including central tendency (e.g. means) or other basic estimates (e.g. regression coefficient) AND variation (e.g. standard deviation) or associated estimates of uncertainty (e.g. confidence intervals)
☐	☒	For null hypothesis testing, the test statistic (e.g. *F*, *t*, *r*) with confidence intervals, effect sizes, degrees of freedom and *P* value noted<br>*Give P values as exact values whenever suitable.*
☒	☐	For Bayesian analysis, information on the choice of priors and Markov chain Monte Carlo settings
☒	☐	For hierarchical and complex designs, identification of the appropriate level for tests and full reporting of outcomes
☒	☐	Estimates of effect sizes (e.g. Cohen's *d*, Pearson's *r*), indicating how they were calculated

Our web collection on statistics for biologists contains articles on many of the points above.

Software and code

Policy information about availability of computer code

Data collection	*Provide a description of all commercial, open source and custom code used to collect the data in this study, specifying the version used OR state that no software was used.*
Data analysis	RNA-Seq data were assembled using Trinity (v. 2.0.6), mapping was performed using Bowtie2 (v2.1.0) and kallisto (v.0.43.1) and annotation using BUSCO. Gene expression data analysis of the RNA-Seq data was performed using the BioConductor packages: edgeR (v. 3.24.3), TopGO software (v. 2.88.0), hclust (v 3.6.0). Additional summarization of the enrichment analysis was performed with ReviGO (http://revigo.irb.hr). Genetic variation analysis was performed on a set of target genes and included the use of MAFFT (v.7.305b), RAxML (Galaxy version 1.0.0) and Arlequin (v.3.5.2.2).

For manuscripts utilizing custom algorithms or software that are central to the research but not yet described in published literature, software must be made available to editors/reviewers. We strongly encourage code deposition in a community repository (e.g. GitHub). See the Nature Research guidelines for submitting code & software for further information.

Data

Policy information about availability of data

All manuscripts must include a data availability statement. This statement should provide the following information, where applicable:

- Accession codes, unique identifiers, or web links for publicly available datasets
- A list of figures that have associated raw data
- A description of any restrictions on data availability

All data generated or analyzed during this study are included in this published article (and its supplementary information files). The RNA-Seq datasets generated for this study are available as raw read data for each library (n = 18) through NCBI BioProject PRJNA496596 (GIVE INFO THAT THEY ARE IN SRA DATABASE??). and the transcriptome shotgun Assembly project at DDBJ/EMBL/GenBank under the accession GHLB01000000. The version described in this paper is the first version, GHLB01000000 (NCBI; BioProject: PRJNA496596). Environmental data are available through the Alaska Ocean Observing System [https://portal.aoos.org/old/gulf-of-alaska#metadata/e25fe1f2-1c98-44f6-856f 5d61c87c0384/project/folder_metadata/24099].

Field-specific reporting

Please select the one below that is the best fit for your research. If you are not sure, read the appropriate sections before making your selection.

☐ Life sciences ☐ Behavioural & social sciences ☒ Ecological, evolutionary & environmental sciences

For a reference copy of the document with all sections, see nature.com/documents/nr-reporting-summary-flat.pdf

Ecological, evolutionary & environmental sciences study design

All studies must disclose on these points even when the disclosure is negative.

Study description	Neocalanus flemingeri CV individuals were collected during a one-week oceanographic cruise (May 2015) from six locations spanning the inner to outer shelf gradient along the Seward Line in the northern Gulf of Alaska and two stations within Prince William Sound. Gene expression profiles were obtained for individual pre-adults (copepodid stage CV) collected at each station (n=18) using RNA-Seq. Functional analysis of gene expression patterns was compared with genetic distance between individuals from different stations and with environmental gradients.
Research sample	The calanoid copepod, Neocalanus flemingeri, was chosen as the target species because of its ecological importance throughout the subarctic Pacific, including the northern Gulf of Alaska and Prince William Sound. Timing of the collection occurred during the spring coinciding with peak biomass of the population in the upper 100 m. The copepodid CV stage was targeted because it represents the stage that is preparing for diapause, which requires the accumulation of lipid stores to meet the energetic needs for dormancy and reproduction.
Sampling strategy	Field collections were performed as part of the Seward Line long-term observation program. Sample size was determined as a balance between spatial coverage (6 stations) and sequencing depth.
Data collection	Samples were collected by Roncalli, Hopcroft and Lenz, using a CalVET net (53-µm mesh) towed vertically from 100 m depth to the surface. Mixed plankton samples were immediately diluted with surface seawater, and maintained at ~5 ºC prior to and during sorting. From each station actively swimming (healthy) N. flemingeri CVs were sorted under the microscope and preserved within 2hrs of the tow in RNAlater Stabilization Reagent (QIAGEN). Temperature, and salinity were measured using SBE 911+ CTD at all stations to the bottom or a maximum depth of 1000 m. The CTD was connected to a SBE32C rosette with 16 Niskin water-sampling bottles used to collect water for chlorophyll a in a 10 m intervals over the upper 50 m. Water samples were filtered serially through 20 µm Poretics polycarbonate filters and onto Whatman GF/F filters under dim light at low pressure. Chlorophyll was then extracted immediately at -20°C in 90% acetone for the two size fractions (< 20 µm and > 20 µm) and read fluorometrically after 24 hrs.
Timing and spatial scale	Samples were collected between May 5 and 10, 2015 at six locations: four stations spanning the inner shelf to outer shelf gradient along the Seward Line in the northern Gulf of Alaska and two stations in adjoining Prince William Sound. The spatial scale ranged from the near-shore nutrient limited region to off shore high nutrient-low chlorophyll (HNLC) waters, with two stations located in the transition region, and two stations in the more productive Prince William Sound. The 6-day sampling period provided comparable time points across the region.
Data exclusions	High quality RNA-Seq data and Individual gene expression profiles were obtained for all 18 individuals. Cluster and genetic analysis showed that a single CV individual from GAK9 station had a lower percentage of reads mapped to the reference than all others (82% vs. 89-95%). While this individual clustered with the other two individuals with respect to gene expression, it also showed genetic divergence from the other individuals. Thus, to avoid bias, this individual was removed from downstream gene expression analysis.
Reproducibility	Because this study involves environmental sampling - each observation is unique and cannot be "reproduced". Returning to the sampling sites in other years cannot duplicate the conditions we observed in 2015. However, RNA-Seq data are available and can be used by others for duplicate or additional analyses.
Randomization	N. flemingeri were sorted from mixed zooplankton samples and Individuals from each station were randomly sorted under the microscope and preserved in RNAlater for RNA extraction.
Blinding	"Blinding" in the traditional sense was not possible for this experiment - however, collections, extractions of samples and gene library preparation/sequencing were performed by different researchers. The researchers who did the bench work did not have prior knowledge about the experimental design. The researchers doing the data analyses worked with station identifications, but had no prior knowledge of environmental conditions at the collection sites.

Did the study involve field work? ☒ Yes ☐ No

Field work, collection and transport

Field conditions	Samples were collected during the May oceanographic cruise as part of the Seward Long-Term Observation Program (LTOP) (http://www.sfos.uaf.edu/sewardline/). The Seward Line is a long-term observation program (LTOP) started in 1998.
Location	The cruise took place in the northern Gulf of Alaska along the Seward Line and in Prince William Sound (http://research.cfos.uaf.edu/sewardline/).

Access and import/export	Collection and handling of the samples were done in compliance with accepted methodologies. Preserved samples were transported following guidelines of shippers (dry ice).
Disturbance	The individuals preserved for this study were obtained in collaboration with an established sampling program, thus, requiring no additional disturbance to the community.

Reporting for specific materials, systems and methods

We require information from authors about some types of materials, experimental systems and methods used in many studies. Here, indicate whether each material, system or method listed is relevant to your study. If you are not sure if a list item applies to your research, read the appropriate section before selecting a response.

Materials & experimental systems

n/a	Involved in the study
☒	☐ Antibodies
☒	☐ Eukaryotic cell lines
☒	☐ Palaeontology
☒	☐ Animals and other organisms
☒	☐ Human research participants
☒	☐ Clinical data

Methods

n/a	Involved in the study
☒	☐ ChIP-seq
☒	☐ Flow cytometry
☒	☐ MRI-based neuroimaging
